# The miR-199a-3p regulates the radioresistance of esophageal cancer cells via targeting the AK4 gene

**DOI:** 10.1186/s12935-018-0689-6

**Published:** 2018-11-16

**Authors:** Chunbao Zang, Fangfang Zhao, Lei Hua, Youguang Pu

**Affiliations:** 1 Department of Radiation Oncology, Anhui Provincial Cancer Hospital, West Branch of the First Affiliated Hospital of USTC, Division of Life Sciences and Medicine, University of Science and Technology of China, Hefei, 230001 Anhui People’s Republic of China; 2 Department of Cancer Epigenetics Program, Anhui Provincial Cancer Hospital, West Branch of the First Affiliated Hospital of USTC, Division of Life Sciences and Medicine, University of Science and Technology of China, Hefei, 230001 Anhui People’s Republic of China; 3 Department of Provincial Clinical College, Anhui Provincial Hospital of Anhui Medical University, Hefei, 230031 Anhui China

**Keywords:** Esophageal cancer, Radioresistance, miR-199a-3p, AK4, TGFβ signaling pathway

## Abstract

**Background:**

MiRNAs was recognized as vital regulators involved in cancer development. Radioresistance remains a major obstacle for effective treatment of cancers. The mechanisms on the miRNA-mediated radioresistance of cancers are still poorly understood. The main subject of this study is to find new miRNA biomarker that regulates the radioresistance of esophageal cancer (EC).

**Methods:**

The cumulative dose of radiation assays were used to screen the EC radioresistant cell lines. Wound-healing and invasion assays were used to characterize the properties of these cell lines. The following survival fraction experiments were performed to test the effects of miR-199a-3p and AK4 in the radioresistance of EC. In addition, we used the luciferase reporter assays to identify the putative underlying mechanism that relates to the miR-199a-3p regulated radio-resistance.

**Results:**

We found that the AK4 gene is one of the targets of miR-199a-3p, which promotes the radioresistance of EC cells. The following experiments by force reversal of the miR-199a-3p or AK4 levels confirmed the relationship of miR-199a-3p and AK4 with the radioresistance of EC cells. In addition, the activities of several signaling pathway were drastically altered by the forced changes of the miR-199a-3p level in EC cells.

**Conclusion:**

Taken together, we found that miR-199a-3p can be potentially used as a biomarker for the EC radioresistance. Moreover, these results provides new insights into the mechanism on the radioresistance of EC cells, and also might guide the clinical therapy of EC.

## Background

MiRNAs (miRNAs) are small non-coding RNAs that function as post-translational regulators of gene expression [1]. In recent years, increasing studies have been focused on the roles of miRNAs in regulating every biological event in normal cells, as well as in cancer cells [2, 3]. Notably, an individual miRNA can have hundreds of targets, while a single target gene may be regulated by many different miRNAs. Evidently, the dysregulation of miRNAs has been found in cancers, and thus the expression profiling of miRNA levels has already been used as diagnostic and prognostic biomarkers to assess tumor development [4]. Moreover, the previous studies demonstrated the roles of various miRNAs in different types of cancers, such as breast, colon, gastric, lung, and prostate [5–7]. Even more importantly, one type of miRNA might also participate in different cancers. For instance, the accumulating studies showed that the dysregulation of miR-199a is found in various cancers, including hepatocellular carcinoma [8], ovarian cancer [9], renal cell carcinoma [10], osteosarcoma [11] and etc. [12, 13]. All these studies demonstrated the complicated networks on miRNA-regulated cancer biogenesis.

Esophageal cancer (EC) is the eighth most commonly occurred cancer worldwide. It has proven to be one of the most difficult malignancies to cure [14, 15]. To date, the chemotherapy or chemoradiotherapy are still the preferred methods for clinical therapy of EC at more advanced stages. However, the chemoresistance and radioresistance are the major obstacles for the effective therapy. Moreover, there is still limited knowledge on the underlying mechanism that governs the chemoresistance and radioresistance of EC. To address this issue, we try to identify new miRNA biomarker that relates to the radioresistance of EC. In our previous studies, we have found that several miRNAs are involved the drug resistance of osteosarcoma by targeting different genes [16–19]. However, whether these miRNAs are involved in the EC radioresistance is still unknown. In this study, using a systematic analysis and profiling methods, we identified that Kyse30-R and Kyse150-R cells are the radioresistant cells of EC. Further investigations in EC cells found that the miR-199a-3p targets AK4, which was reported to be involved in stress, drug resistance, malignant transformation in cancer [20–22]. Taken together, our findings provide a new mechanistic insight into EC radioresistance, which might give us hints for a rational design of the clinical therapy against EC.

## Methods

### Cells and culture

The human esophageal cancer Cells lines Kyse30 and Kyse150 were kindly provided by Professor Zhan (National Laboratory of Molecular Oncology, China, Beijing) [23, 24], Kyse30-R and Kyse150-R were obtained from their parental strains of Kyse30 and Kyse150, respectively. Four cells were cultured and maintained in RPMI medium 1640 (Biological Industries) supplemented with 10% fetal bovine serum (PAN Biotech), 100 U/ml penicillin, and 100 mg/ml streptomycin (WISENT INC) in humidified air at 37 °C with 5% CO_2_.

### Transient transfection assays

The Homo sapien miR–199a-3p mimic, antagomiR and scrambled negative control (NC) were obtained from Guangzhou Ribobio, China. All the transfection experiments were performed using riboFECT CP transfection kit were supplied by Guangzhou Ribobio, China. Western blot and qRT–PCR assays were performed to confirm the effect of AK4 on the expression of miR–199a-3p. The sequences used in this study are as follows:

si-AK4:

GCCTAATGATGTCCGAGTT

5′-GCCUAAUGAUGUCCGAGUU dTdT-3′

3′-dTdT CGGAUUACUACAGGCUCAA-5′

### Reverse transcription-quantitative polymerase chain reaction (qRT-PCR) assays

Total RNA was extracted from cells using TRIzol reagent (Tiangen) according to the manufacturer’s instructions. The reverse-transcription and PCR primers for miR-199a-3p and U6 were purchased from GenePharma. The cDNA library was synthesized using the PrimeScript RT reagent kit (Tiangen). The mRNA expression level of AK4 using TaqMan assay and the miRNA using SYBR Green assay (Biosystems) were quantified in an FTC-3000PCR instrument (Funglyn). Either U6 small nuclear RNA (HmiRQP9001) or β-actin (ShingGene) were used as an internal control. Expression levels were calculated using the relative quantification method (2^−∆∆^Ct). Each test was repeated in triplicate. The sequences of the primers and probes used for the qRT-PCR analysis are:

hAK4 F: 5′-CACTTCTTGCGGGAGAACATC-3′

hAK4 R: 5′-CCAACTCGGACATCATTAGGC-3′

hAK4 probe: 5′-FAM-CAGCACCGAAGTTGGTGAGATGGC-3′

hACTB F: 5′-GCCCATCTACGAGGGGTATG-3′

hACTB R: 5′-GAGGTAGTCAGTCAGGTCCCG-3′

hACTB probe: 5′CY5-CCCCCATGCCATCCTGCGTC-3′

### Radiation exposure and clonogenic assays

All cells were pretreated by NC, miR-199a-3p mimics, antagomiRs and si-AK4 for 24 h, then were digested and counted according to 0 Gy (500), 2 Gy (1000), 4 Gy (2000), 6 Gy (5000), 8 Gy (8000) cells/well and was inoculated in a 6-well plate in triplicate, the corresponding dose was irradiated after 24 h, using a 6-MV X-ray generated by a linear accelerator (varian trilogy at a dose rate of 2 Gy/min) and the culture was continued for 15 days, then washed and fixed with 10% formaldehyde, and giemsa stained. The number of cloned spheres with > 50 cells was counted, and the number of cells inoculated with 50–200 cloned spheres was selected as the appropriate number of colonies for colony formation experiments. The overall experiment was repeated 3 times and the mean was taken. Calculate the cell clone formation rate (planting efficiency, PE = number of cloned cells/number of cells inoculated × 100%) and cell survival fraction (SF = each dose of PE/non-irradiated PE × 100%), using the multi-target click model of GraphPad Prism 6 software (GraphPad), The cell dose survival curve was fitted according to the formula SF = 1 × (1 − e − D/D0)^N^, and the radiosensitivity parameters (D0, N, Dq and SF2).

### Wound-healing assays

For cell motility assays, cells were grown to near confluence in 24-well plates in full-growth medium and were then incubated overnight in serum-free medium. Cells were scratched with a 10 μl sterile pipette tip and extensively washed with PBS to remove cells debris. Cells were then incubated in medium containing 10% FBS. The wounded areas were photographed and measured after scratching 0, 8, 12, 16, 20, 24, using a CKX41 inverted microscope (Olympus).

### Invasion assays

Invasion assays were conducted in a 24-well plate with 8 μm pore size membranes Matrigel-coated Transwell chambers (Corning). 3 × 10^4^ cells were seeded into the upper chambers in 200 μl serum-free RPMI-1640, while 600 μl RPMI-1640 supplemented with 10% fetal bovine serum was placed in the lower chamber. After incubation for 36 h at 37 °C and 5% CO_2_, the invasion potential of the cells that moved to the lower surface invading 8 μm pore size membranes with Matrigel were fixed with 70% ethanol and stained with 0.1% crystal violet for 30 min. The cells were then imaged and counted in five random fields using a CKX41 inverted microscope (Olympus). Each test was repeated in triplicate.

### Cell proliferation assay

The capacity for cellular proliferation was measured by CCK8-based cell proliferation assay. Cells were seeded in 96-well plates at a density of 5 × 10^3^ cells per well, and cell proliferation assays were performed every 24 h using CCK8. The number of viable cells was measured by their absorbance at 450 nm at the indicated time points.

### Drug resistance profiling

For cell proliferation assay, cells in the logarithmic phase of growth were seeded in triplicate in 96-well plates at a density of 4 × 10^3^ cells/well and treated with 4-fold serially diluted drugs for 72 h. Then, 10 μl of CCK8 salt (Bimake) was added to the corresponding well, the cells were incubated at 37 °C for an additional 2 h. The optical density was determined with a microplate reader (TECAN) at a wavelength of 450 nm.

### Western blotting assays

Cells protein lysates were separated by 10% SDS-poly acrylamide gel electrophoresis (SDS-PAGE), transferred to 0.45 μm PVDF Transfer Membranes (Immobilon^®^-P). Next, the PVDF membrane was blocked with 5% non-fat dairy milk in phosphate-buffered saline (PBS) with 0.1% Tween-20. The first antibodies were then detected by second antibodies, which could recognize them conjugated to enzyme horseradish peroxidase. The information of antibodies were as follows: anti-rabbit (San Ying Biotechnology, China), anti-mouse (SanYing Biotechnology, China), anti-GAPDH (San Ying Biotechnology, China), the rabbit polyclonal antibody of AK4 was bought from Proteintech (AP20571a), the concentration was 45 μg/150 μl. The target bands were visualized by an enhanced chemiluminescence reaction (Pierce), and the relative band intensity was determined by the Gel-Pro Analyzer 4.0 software (Media Cybernetics).

### Luciferase reporter assays

The AK4 wild-type (WT) 3′UTRs, which contain the putative miR-199a-3p binding site, were cloned into the pEZX-MT01-luciferase-report vector (GeneCopoeia™). For the luciferase reporter assay, Kyse30 and Kyse30-R cells were co-transfected with a luciferase reporter vector and negative control, the miR-199a-3p mimic or antagomiR. After 24 h transfection, the cells were assayed for luciferase activity using the Dual-Luciferase Reporter Assay System (Promega) in a Promega GloMax 20/20 luminometer, according to the manufacturer’s instructions. The relative firefly luciferase activities of the 3′UTR and pathway reporter vector were analyzed as previously reported [25]. All experiments were repeated in triplicate.

### Signaling pathway analysis

Constructs for the reporters of seventeen signaling pathways were obtained from SABiosciences (USA) and analyzed according to the manufacturer’s instructions. The cells were transfected in triplicate with each firefly luciferase reporter construct in combination with the Renilla luciferase-based control construct using transfection reagent, and both the luciferase activities were measured in the cell extracts 48 h after transfection. The luciferase activities (luciferase unit) of the pathway reporter relative to those of the negative control in the transfected cells were calculated as a measurement of the pathway activity.

### Statistical analyses

The data are presented as the mean ± standard deviation. All statistical analyses were conducted by Excel and GraphPad Prism 6. Statistical significance was assessed by a two-tailed unpaired Student’s *t*-test, a one-way analysis of variance or Mann–Whitney U test. Results were considered to be statistically significant at p < 0.05.

## Results

### Characterization of Kyse30-R and Kyse150-R cells as radioresistant strains of esophageal cancer

**Fig. 1:**
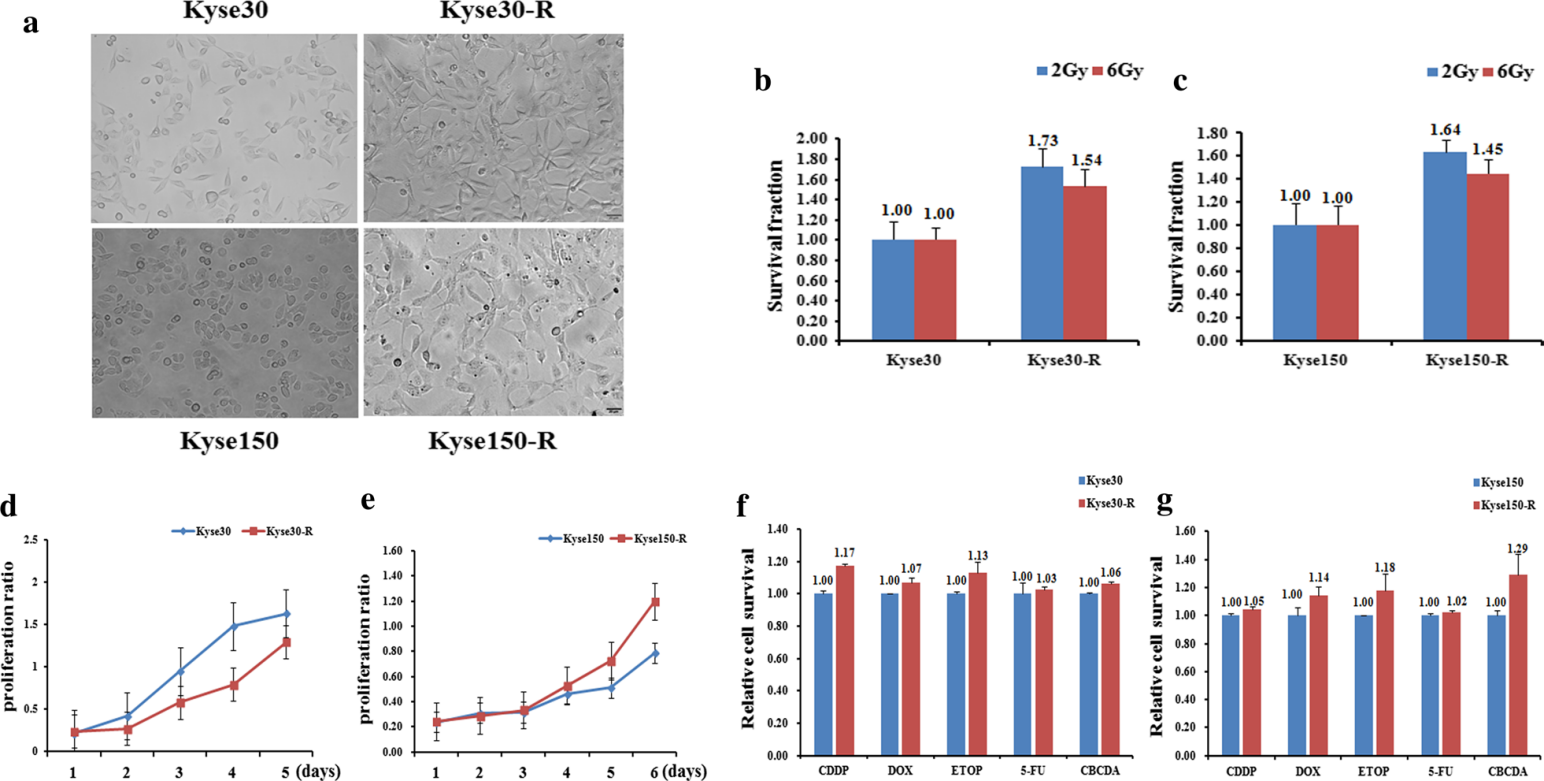
Establishment, identification and biological characteristics of radiotherapy resistant strains of esophageal cancer cells. **a** Cell morphology identification. Kyse30-R and Kyse150-R cell lines were established from Kyse30 and Kyse150, respectively. The cumulative dose of radiation of Kyse30-R and Kyse150-R reached to 70 and 82 Gy, respectively. Under the optical microscope, the morphology of the cell line was obviously changed. Most of the cell lines were long spindle shaped and protruding, and the cell space was enlarged and the parental cells were mostly oval and cobblestone, and the cells were closely connected. **b**, **c** Radiosensitivity detection assay showed that the sensitivity of Kyse150-R and Kyse30-R cells was lower than that of parental cells. **d**, **e** Cell proliferation assay showed that the proliferation of Kyse30-R cells was slower than that of parental cells, however, the proliferation activity of Kyse150-R cells was stronger than that of parental cells. **f**, **g** Chemosensitivity and resistance assay showed that Kyse150-R and Kyse30-R cells were drug resistance than that of their parental cells

To underlie the characteristics of the radioresistance of EC cells, we first aim to screen the radioresistant strains of EC. We thus used the parental strains of Kyse30 and Kyse150 and make them subjected to X-ray radiation at increasing doses. After several rounds of screening against X-ray challenge, we successfully obtained two mutated EC strains that are radioresistant compared to the parental strains. They can tolerate the X-ray radiation at a dose up to 70 and 82 Gy, respectively, we thus termed them as Kyse30-R and Kyse150-R respectively. During the X-ray challenge, the morphology of the cell lines was obviously changed. Compared to the oval shape of the parental cells, most of the Kyse30-R and Kyse150-R cells were long spindle shaped and protruding outwards to connect with each other. The cell volume was also somewhat enlarged for the radioresistant cell lines (Fig. 1a). The following radiosensitivity detection assays confirmed that Kyse30-R and Kyse150-R are more resistant against radiation (Fig. 1b, c). In addition, we performed the cell proliferation experiment to further characterize these two cell lines. The results showed that the cell proliferation capability of Kyse30-R cells was weaker than that of parental cells, whereas the Kyse150-R cells showed stronger cell proliferation activity than that of parental cells (Fig. 1d, e). Next, we performed the drug-resistance profiling of the two cell lines against the following drugs: CDDP, Dox, ETOP, 5-FU, CBCDA (Fig. 1f, g). In agreement with the radioresistance profiles, these two cell lines are also a little bit more resistant against the above drugs, which indicates that Kyse30-R and Kyse150-R cells are also chemoresistant cells compared to the parental cells.

**Fig. 2:**
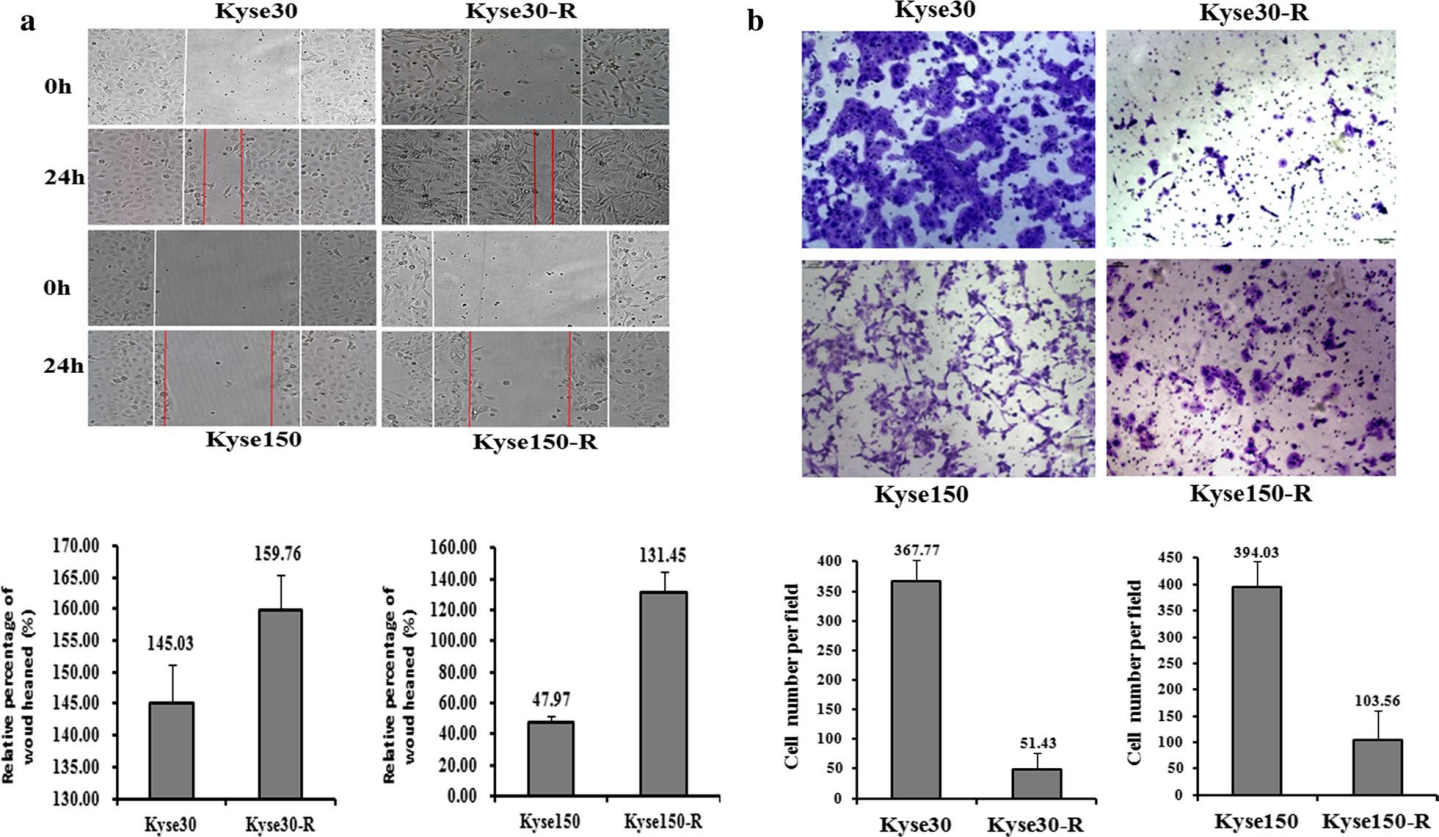
The migration and invasion ability between radiotherapy resistant strains and parental cells. **a** The migration ability of Kyse30-R and Kyse150-R cells is higher than that of parental cells. **b** The invasion ability of Kyse30-R is weaker than that of parental cells, and the invasion ability of Kyse150-R cells was higher than that of parental cells

Generally, the cancer cells are featured with the higher ability of cell migration and invasion. So we detected the migration and invasion ability of resistant cells Kyse30-R and Kyse150-R to see whether they obtained the characteristics of cancer cells. As shown in Fig. 2a, the wound healing assays showed that the cell migration abilities of Kyse30-R and Kyse150-R cells are dramatically increased, as compared to the parental cells. Furthermore, the Kyse150-R cells also showed higher invasion ability compared to the parental cells (Fig. 2b). By contrast, the invasion ability of Kyse30-R cells is lower than that of the parental cells (Fig. 2b). These results demonstrated that the Kyse150-R cells acquired some of the features of the cancer cells, whereas the biological features of Kyse30-R cells is different from the cancer cells. This also indicates that the biogenesis of the cancer cells is rather a complicated process, induced by diverse factors.

### The AK4 is the target of miR-199a-3p

**Fig. 3:**
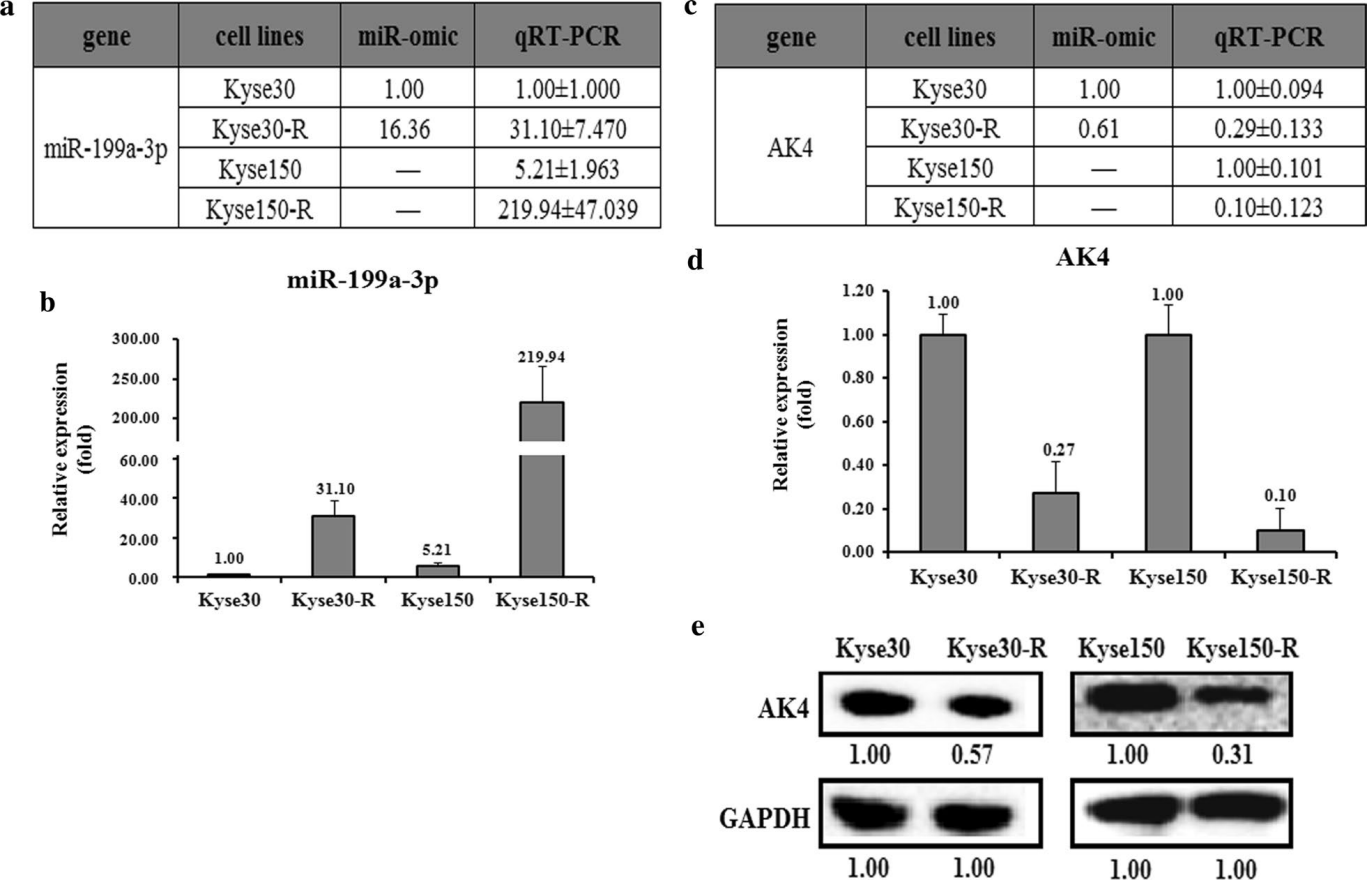
The AK4 level is lower in radiotherapy resistant strains than in parental cells. The relative miR-199a-3p level (fold) in Kyse30-R and Kyse150-R cells versus Kyse30 and Kyse150 cells measured by both miR-omic and qRT-PCR analyses is shown in a table (**a**) and those measured by qRT-PCR are shown in a plot (**b**). The relative level (fold) of the AK4 gene in Kyse30 and Kyse150 cells versus Kyse30-R and Kyse150-R cells are summarized in a table (**c**), with a plot showing the miR-omic and qRT-PCR analyses (**d**) and a figure showing the western blot analysis (**e**). “–” indicates no detection in the omic analysis

MiRNAs usually down-regulate the target genes to fulfill their functions. To see whether miRNAs participate in the process of making the parental cells to be resistant, we select miR-199a-3p as our target for further studies. We first predicted the targets of miR-199a-3p using the following websites: Targetscan and miRDB. Among them, we choose the AK4 gene as our target, which was previously found to be related to cancer drug resistance [26, 27]. We then detected the expression levels of miR-199a-3p and AK4 in the radiosensitive and radioresistant cell lines. The miR-omic and qRT-PCR analyses in Kyse30-R cells versus Kyse30 cells showed that the expression of miR-199a-3p is significantly higher in Kyse30-R cells, resulting in about 16 and 31 folds for the miR-omic and qRT-PCR assays, respectively (Fig. 3a, b). Similarly, the qRT-PCR assay also showed a higher level of Kyse150-R cells compared to the parental cells (Fig. 3a, b). Next we checked the level of AK4 in both the mRNA and protein levels. As shown in Fig. 3c, d, the AK4 mRNA level is reversely correlated with the expression of miR-199a-3p, which means that the AK4 mRNA level is relatively lower in the radioresistant cells (Fig. 3c, d). Consistently, the protein level of AK4 is also down-regulated in Kyse30-R and Kyse150-R cells, with the ratio of about 0.57 and 0.31, respectively (Fig. 3e).

**Fig. 4:**
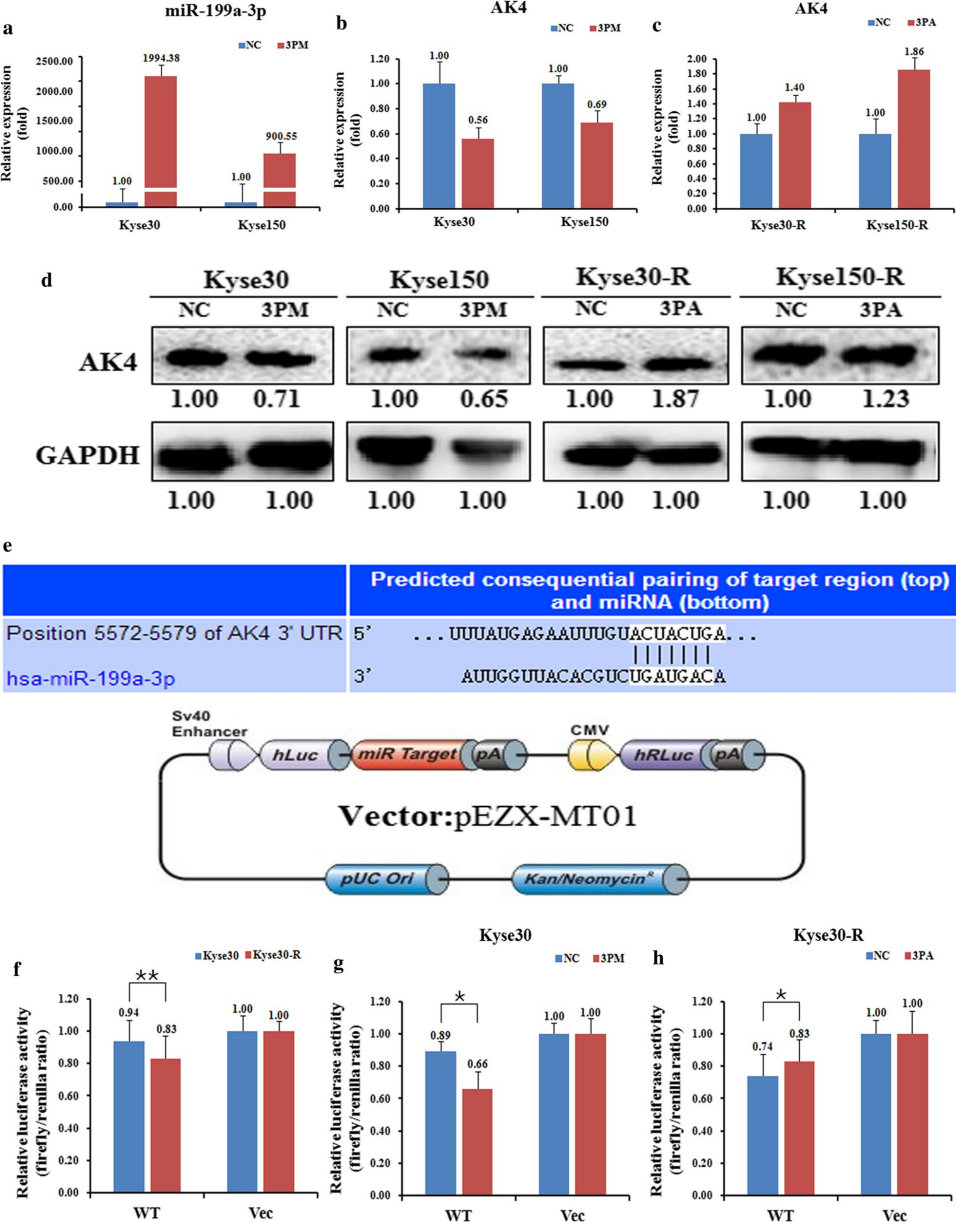
AK4 is a target of miR-199a-3p in esophageal cancer cells. Level of miR-199a-3p (**a**). AK4 mRNA (**b**, **c**) and protein (**d**) levels in the miR-199a-3p mimic (3PM)-transfected Kyse30 and Kyse150 cells and the miR-199a-3p antagomiR (3PA)-transfected Kyse30-R and Kyse150-R cells versus the negative control (NC) cells, as determined by qRT-PCR or western blot analyses. **e** Sequences in the UTR region of the AK4 gene targeted by miR-199a-3p, with the hatched section showing the combined area and the diagram of the vector. The relative luciferase activities (fold) of the reporter with the wild-type (WT) AK4-UTR or without the UTR (Vec) were determined in the EC cells transfected with the miR-199a-3p mimic (in Kyse30), antagomiR (in Kyse30-R) or Mock (**f**–**h**) sequences. The Renilla luciferase activity of a co-transfected control plasmid was used as a control for the transfection efficiency. The representative results from three independent experiments are shown. *p value < 0.05, **p value < 0.01 by Student’s *t*-test

Considering that the AK4 level is negatively correlated with the miR-199a-3p level, we propose that AK4 might be a direct target of miR-199a-3p. We thus changed the miR-199a-3p level by transfecting miR-199a-3p mimic (3PM) into the Kyse30 and Kyse150 cells or the miR-199a-3p antagomiR (3PA) into the Kyse30-R and Kyse150-R cells. As expected, the transfection of 3PM into the Kyse30 and Kyse150 cells indeed increased the miR-199a-3p level to about 1994 and 900 folds, respectively (Fig. 4a). This conflict indicates that 3PA might cause the dysregulation of other factors that contribute to the up-regulation of miR-199a-3p level in the Kyse150-R cells. Following the changes of the miR-199a-3p, the AK4 level in reversely correlated with the changes of miR-199a-3p, resulting in a much lower AK4 level in Kyse30 and Kyse150 cells whereas a higher AK4 level in Kyse30-R and Kyse150-R cells (Fig. 4b, c). Consistently, the AK4 protein level also showed a similar changing trend to the AK4 mRNA level in these four cell lines (Fig. 4d). Next, to further validate AK4 is indeed the target of miR-199a-3p in EC cells, we constructed a reporter vector pZEX-AK4-UTR WT by the fusion of the 3′-untranslated region (UTR) of the AK4 gene harboring the putative binding site of miR-199a-3p with the *Renilla* luciferase gene (Fig. 4e). The construct was transfected into Kyse30 and Kyse30-R cells to test its effect. We found that pZEX-AK4-UTR WT led to a significantly higher luciferase activity in Kyse30 cells than that in Kyse30-R cells (Fig. 4f). Furthermore, following the increase of the miR-199a-3p level, the activity of mimic-transfected Kyse30 cells is dramatically decreased whereas a reverse effect was found for the antagomiR-transfected Kyse30-R cells (Fig. 4g, h). All these results suggested that AK4 is indeed a target of miR-199a-3p in EC cells.

### MiR-199a-3p and AK4 expression are related with the radioresistance of EC cells

**Fig. 5:**
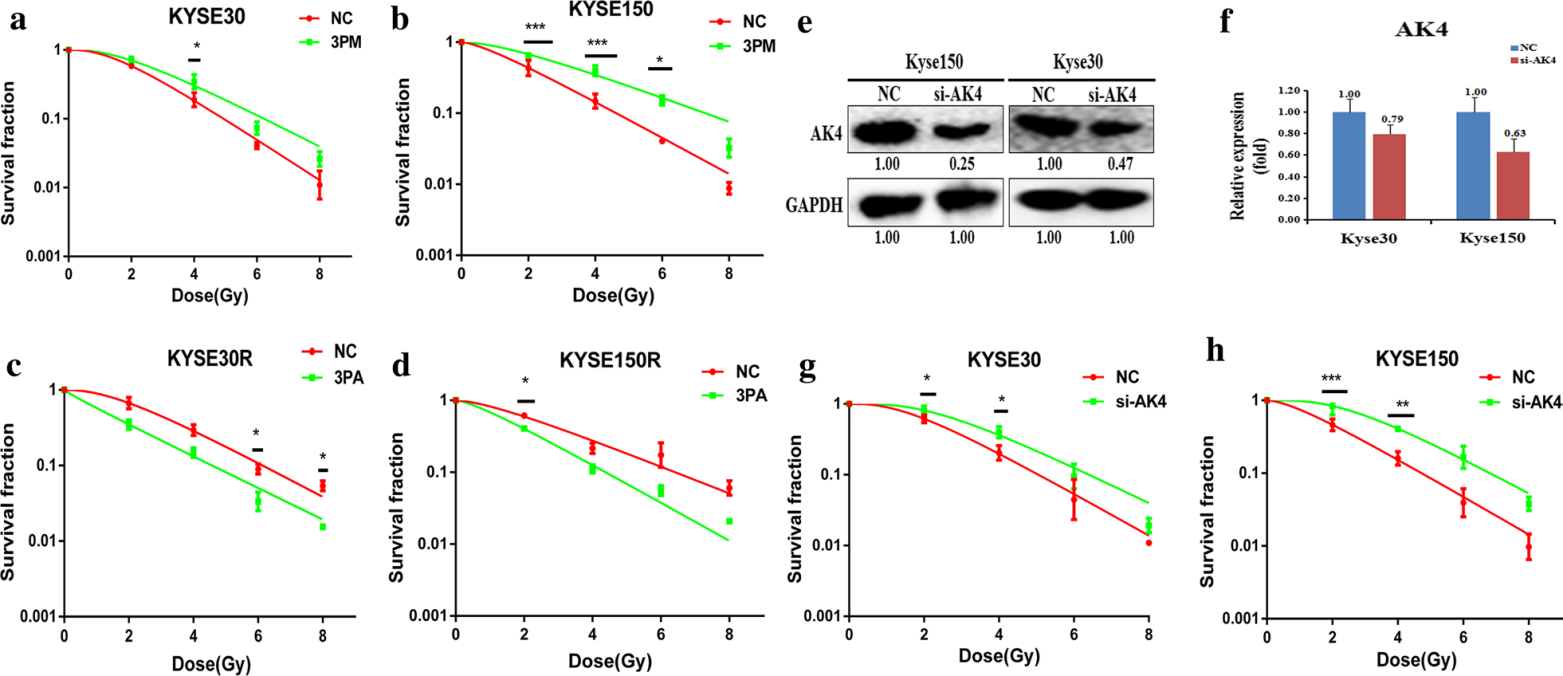
Effects of a forced reversal of the miR-199a-3p or AK4 levels on the esophageal cancer cells. The cells were transfected for 24 h, then cells were digested and counted according to 0 Gy (500), 2 Gy (1000), 4 Gy (2000), 6 Gy (5000), 8 Gy (8000) cells/well and was inoculated in a 6-well plate in triplicate, the corresponding dose was irradiated after 24 h, using a 6-MV x-ray generated by a linear accelerator Varian trilogy at a dose rate of 2 Gy/min (varian trilogy at a dose rate of 2 Gy/min). **a**, **b** MiR-199a-3p mimic (3PM)-transfected Kyse30 and Kyse150 increases survival fraction versus the negative control (NC) cells. **c**, **d** MiR-199a-3p antagomiR (3PA)-transfected Kyse30-R and Kyse150-R decreases NC cells survival fraction versus the negative control (NC) cells. AK4 protein level (western blot analysis) and mRNA determined by qRT-PCR in the si-AK4-transfected versus the NC-transfected Kyse30 and Kyse150 cells **e**, **f**. Si-AK4-transfected Kyse30 and Kyse150 cells increases NC cells survival fraction versus the negative control (NC) cells **g**, **h**. The surviving fraction was calculated using the multitarget single-hit model: Y = 1 − (1 − exp(− k * x))^N^. The data are presented as the mean ± standard deviation of results from 3 independent experiments, and two way anova was used to calculate statistical significance

We found that AK4 and miR-199a-3p are the differentially expressed targets in EC cells, and miR-199a-3p negatively regulates the expression of AK4. To see whether AK4 and miR-199a-3p are related to the radioresistance of EC cells, we compared the effect on drug-triggered cell death in different EC cell lines. The transfection of miR-199a-3p mimic into Kyse30 or Kyse150 cells increased the cell survival rate against radiation (Fig. 5a, b). Reversely, transfection of miR-199a-3p antagomiR into Kyse30-R or Kyse150-R cells somewhat decreased the cell survival rate against radiation (Fig. 5c, d). These results suggest that miR-199a-3p positively correlates with the radioresistance of EC cells. Next we down-regulates the expression of AK4 by transfection of si-AK4 into Kyse30 or Kyse150 cells. Western blot and qRT-PCR analysis showed that the expression of AK4 is significantly down-regulated upon the transfection of si-AK4 (Fig. 5e, f). The resultant radioresistant assays showed that down-regulation of AK4 increased the cell survival capability against radiation, which means that AK4 suppresses the radioresistance of EC cells (Fig. 5g, h).

### MiR-199a-3p regulates the activity of the TGFβ signaling pathway in the context of EC radioresistance

**Fig. 6:**
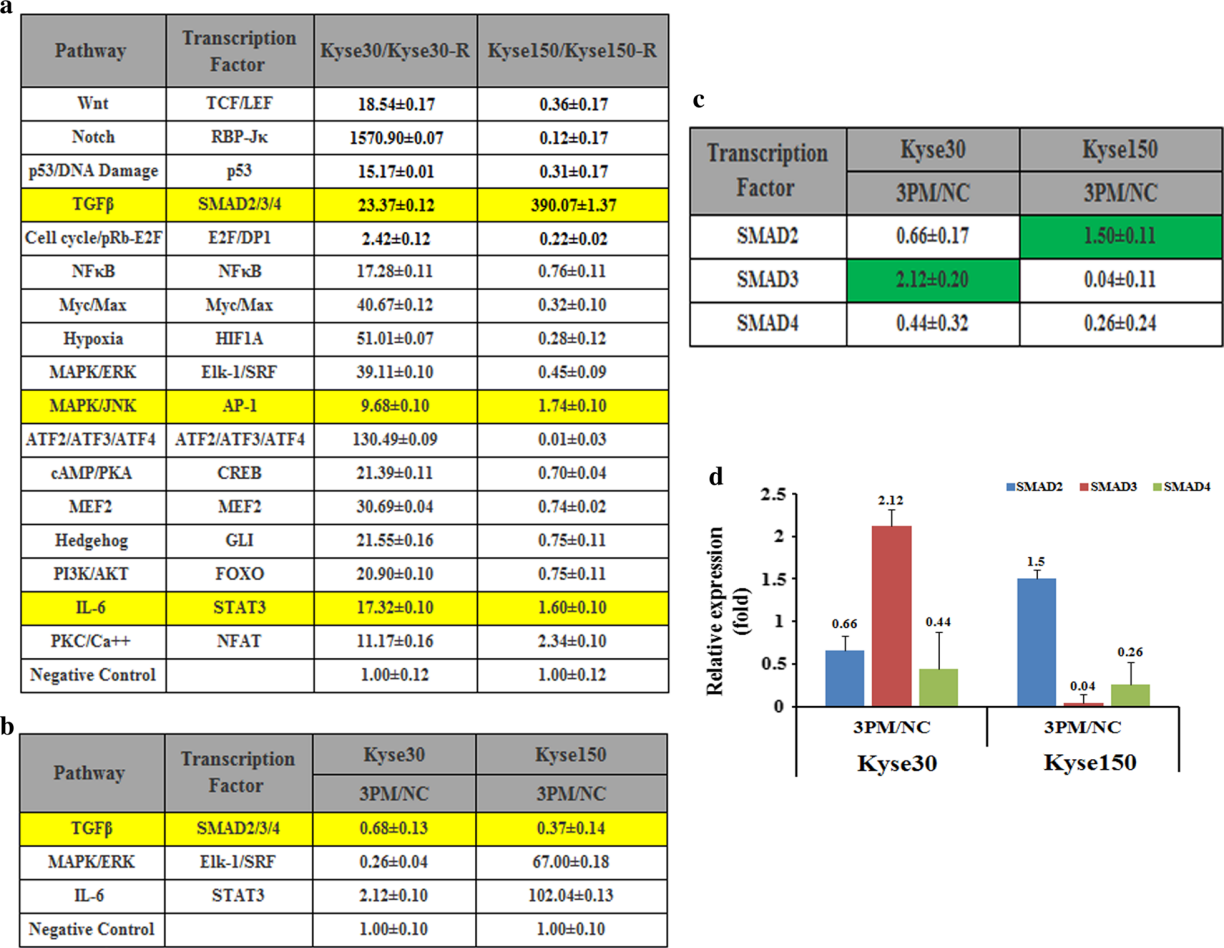
The effects of the forced reversal of miR-199a-3p levels on the activity of the signaling pathways on the esophageal cancer cells. **a** Relative activities (mean ± SD) of the seventeen pathways in Kyse30, Kyse150 Kyse30-R and Kyse150-R cell lines. Three pathways of TGFβ, MAPK/JNK and IL-6 with consistent change trend between Kyse30/Kyse30-R and Kyse150/Kyse150-R. **b** The relative pathway activities of the three pathways in the AK4 siRNA versus the NC-transfected Scaber and Kyse30, Kyse150 cells. The relative expression ratio of the four transcription factors in the AK4 siRNA versus the NC-transfected Scaber and Kyse30, Kyse150 cells by qRT-PCR analyses were shown in Table (**c**) and in plot (**d**) (NC was normalized)

To further elucidate the underlying mechanism of EC radioresistance mediated by miR-199a-3p, we compared the activities of seventeen cancer-related signaling pathways in both Kyse30-R and Kyse150-R versus their parental cells Kyse30 and Kyse150, respectively (Fig. 6a). Notably, all of the tested signaling pathways are up-regulated in the Kyse30 cells, as compared to the resistant cells Kyse30-R. By contrast, most of signaling pathways are down-regulated in the Kyse150 cells. Only three pathways: TGFβ, MAPK/JNK and IL-6 are up-regulated in the Kyse150 cells, which are in agreement with that of Kyse30 cells. We thus take these three pathways for further studies. We tested the expression level of these three pathways by forced changes in the miR-199a-3p level in both Kyse30 and Kyse150 cells. Upon the transfection of the miR-199a-3p mimic into Kyse30 cells, the activities of TGFβ and MAPK/JNK were down-regulated whereas that of IL-6 was up-regulated accompanied by the elevation of the miR-199a-3p level (Fig. 6b). Similar effect was also found for the TGFβ pathway in the Kyse150 cells upon the transfection of miR-199a-3p mimic. Altogether, only the TGFβ pathway was proposed to be involved in the forced changes of miR-199a-3p level. Generally, the TGFβ pathway was regulated by three transcription factors, SMAD2/3/4, we thus tested the individual levels of SMAD2/3/4 upon the transfection of 3PM in the Kyse30 and Kyse150 cells. The results showed that only SMAD4 is down-regulated in both Kyse30 and Kyse150 cells (Fig. 6c, d). The results demonstrated that the TGFβ signaling pathway might be involved in the miR-199a-3p-regulated EC radioresistance. However, further investigations are needed to further confirm the relationship between AK4 and the TGFβ signaling pathway.

## Discussion

MiRNAs play vital roles in various biological processes such as proliferation, apoptosis and differentiation, via regulating gene expression at post-modification level [28]. Accumulating evidences have suggested that miR-199a-3p is involved in cancer biology [29, 30]. Moreover, miR-199a showed distinct expression profiles in several types of cancer [31, 32]. For instance, miR-199a-3p is downregulated in hepatocellular carcinoma, resulting in an increased sensitivity to doxorubicin-induced apoptosis [33]. Down-regulation of miR-199a-3p in cisplatin-resistant breast cancer is able to attenuate cisplatin resistance via regulating the mitochondrial transcription factor A [34]. All these studies indicated that miR-199a-3p may be involved in cancer chemotherapy resistance. In accordance with previous findings, here we showed that miR-199a-3p also involves in EC radioresistance. The results described here increased the knowledge of miR-199a-3p on radioresistance of cancer cells. The multi-functional roles of miR-199a-3p in different types of cancers also indicate miR-199a-3p has a potential to be a biomarker for cancer therapy.

We found that the AK4 gene is a target of miR-199a-3p that positively correlates with the EC radioresistance. AK4 was reported to be involved in the development of cancers, and is used as a potential therapeutic target for anticancer treatment. For example, the AK4 expression level could modulate the anti-cancer drug sensitivity through regulating mitochondrial activity [26]. Of note, a previous study found that AK4 promotes the metastasis of lung cancers by down-regulating the transcription factor ATF3 [21]. In agreement with the previous findings, here we demonstrated that the expression level of AK4 is associated with the EC radioresistance, which might be regulated by miR-199a-3p. However, the fine mechanism for the miR-199a-3p/AK4-mediated EC radioresistance remains to be elucidated.

## Conclusions

In this work, we screened Kyse30-R and Kyse150-R EC radioresistant cells, and find miR-199a-3p/AK4-mediated EC radioresistance, the findings suggest that miR-199a-3p or AK4 may serve as biomarkers for the potential therapeutic treatment of EC.

## Abbreviations

EC: esophageal cancer
miR: microRNA
AK4: adenylate kinase 4
3PA: miR-199a-3p antagomiR
3PM: miR-199a-3p mimic
UTR: untranslated region
WT: pEZX-MT01-AK4 UTR wild type
Vec: pEZX-MT01 enhancer control
PBS: phosphate-buffered saline
CDDP: cisplatin
Dox: doxorubicin
Etop: etoposide
5-FU: 5-fluorouracil
CBCDA: carboplatin
